# Medium Chain Acylcarnitines Dominate the Metabolite Pattern in Humans under Moderate Intensity Exercise and Support Lipid Oxidation

**DOI:** 10.1371/journal.pone.0011519

**Published:** 2010-07-12

**Authors:** Rainer Lehmann, Xinjie Zhao, Cora Weigert, Perikles Simon, Elvira Fehrenbach, Jens Fritsche, Jürgen Machann, Fritz Schick, Jiangshan Wang, Miriam Hoene, Erwin D. Schleicher, Hans-Ulrich Häring, Guowang Xu, Andreas M. Niess

**Affiliations:** 1 Central Laboratory, Division of Clinical Chemistry and Pathobiochemistry, University Hospital Tuebingen, Tuebingen, Germany; 2 CAS Key Laboratory of Separation Science for Analytical Chemistry, Dalian Institute of Chemical Physics, Chinese Academy of Sciences, Dalian, China; 3 Department of Sports Medicine, Disease Prevention and Rehabilitation, Johannes Gutenberg-University Mainz, Mainz, Germany; 4 Institute for Clinical and Experimental Transfusion Medicine, University Hospital Tuebingen, Tuebingen, Germany; 5 Immatics biotechnologies GmbH, Tuebingen, Germany; 6 Section on Experimental Radiology, University Hospital Tuebingen, Tuebingen, Germany; 7 Department of Internal Medicine 4, University Hospital Tuebingen, Tuebingen, Germany; 8 Department of Sports Medicine, University Hospital Tuebingen, Tuebingen, Germany; McMaster University, Canada

## Abstract

**Background:**

Exercise is an extreme physiological challenge for skeletal muscle energy metabolism and has notable health benefits. We aimed to identify and characterize metabolites, which are components of the regulatory network mediating the beneficial metabolic adaptation to exercise.

**Methodology and Principal Findings:**

First, we investigated plasma from healthy human subjects who completed two independent running studies under moderate, predominantly aerobic conditions. Samples obtained prior to and immediately after running and then 3 and 24 h into the recovery phase were analyzed by a non-targeted (NT-) metabolomics approach applying liquid chromatography-qTOF-mass spectrometry. Under these conditions medium and long chain acylcarnitines were found to be the most discriminant plasma biomarkers of moderately intense exercise. Immediately after a 60 min (at 93% V_IAT_) or a 120 min run (at 70% V_IAT_) a pronounced, transient increase dominated by octanoyl-, decanoyl-, and dodecanoyl-carnitine was observed. The release of acylcarnitines as intermediates of partial β-oxidation was verified in skeletal muscle cell culture experiments by probing ^13^C-palmitate metabolism. Further investigations in primary human myotubes and mouse muscle tissue revealed that octanoyl-, decanoyl-, and dodecanoyl-carnitine were able to support the oxidation of palmitate, proving more effective than L-carnitine.

**Conclusions:**

Medium chain acylcarnitines were identified and characterized by a functional metabolomics approach as the dominating biomarkers during a moderately intense exercise bout possessing the power to support fat oxidation. This physiological production and efflux of acylcarnitines might exert beneficial biological functions in muscle tissue.

## Introduction

Physical activity has notable health benefits for the general population and is an economical and efficient intervention for treating metabolic syndrome and type 2 diabetes [1]–[3] as well as for preventing the associated increase in morbidity and mortality. The adaptation of the skeletal muscle to endurance training can be described as an increase in the capacity and efficiency to utilize fuels [4]–[6], predominantly fat and carbohydrates for the generation of ATP [7], [8]. Furthermore, the working muscle produces and releases substances during exercise which not only mediate the adaptation of the muscle, but also improve the metabolic flexibility of the complete organism leading to adjustable substrate utilization, e.g. with improved oxidation of fat and decreased demand for glucose and glycogen [9]–[11]. Skeletal muscle secretes peptides and metabolites that participate in the regulation of whole body glucose and lipid homeostasis. Recently, the group of BK Pedersen introduced the term myokines for such cytokines and other peptides that are produced, expressed, and released by muscle fibers and exert either paracrine or endocrine effects [12]. The discovery of C16:1n7-palmitoleate as an insulin-sensitizing lipokine revealed the existence of a network which uses metabolites to communicate with other organs [13].

Metabolomics approaches encompass the targeted and non-targeted, comprehensive analysis of metabolites which represent powerful tools for the investigation of complex metabolic processes [14]–[18]. A limited number of metabolomic studies on specific aspects of exercise in humans, using gas chromatography-time of flight (TOF) mass spectrometry (MS) have been reported to date, including effects of strenuous cycling [19], training associated metabolic changes in professional rowers [20], the effects of nutritional modification on the metabolome during the recovery phase [21] and the comparison of urinary pattern of trained and untrained women by ^1^H-NMR [22].

Here, we applied a non-targeted (NT-) metabolomics approach to investigate the changes in plasma metabolite profile immediately after moderately intense exercise and following 3 h and 24 h of recovery, to identify exercise-related biomarkers, and to explore their potential biological function. We studied a profound metabolic challenge under physiological metabolic conditions in a study group consisting of young, healthy men. Two groups completed an exercise protocol of continuous endurance running on a treadmill for 60 or 120 min at moderate intensity. Using liquid chromatography-qTOF-mass spectrometry, medium and long chain acylcarnitines were identified as the most distinct exercise biomarkers at the different time points with a transient, up to nine-fold increase immediately after the run. Moreover, we provide evidence for a positive biological function of extracellular acylcarnitines on lipid oxidation demonstrated in human myotubes and skeletal muscle tissue.

## Results

### Standard laboratory parameters of the first study

In the first study, 13 lean, healthy individuals (table 1) performed a treadmill run for 60 min at a speed which corresponded to 93% velocity at the individual anaerobic threshold (V_IAT_; approx. 75% VO_2max_) resulting in significantly increased blood lactate concentrations after 10 min of exercise performance and thereafter (Table 2), but remaining in a range typical for moderate, predominantly aerobic exercise [23]. Plasma somatotropin levels (13.7±8.2 vs. 1.13±1.8 µg/L) and norepinephrine levels (4.28±1.37 vs. 1.88±0.71 nmol/L) were significantly increased after running, while epinephrine, creatine kinase activity, and cortisol plasma concentrations were not elevated (Table 2). The significant decrease of cortisol after 3 h of recovery phase is likely to represent the circadian decline around 1 pm. Moreover, treadmill running resulted in strongly increased plasma nonesterified free fatty acids levels (NEFA; 1276±392 versus 395±115 µmol/L). Plasma samples of the first study were used for the NT-metabolomics analyses.

**Table 1:** Physical characteristic of the subjects.

	first exercise study(n = 13)		second exercise study(n = 8)	
	Mean	SE	mean	SE
Age (yrs)	32.6	±6.1	30.9	±5.8
Body mass index (kg×m^2^)	21.6	±1.2	21.8	±1.2
VO_2_ max (ml/kg/min)	56.5	±1.4	63.0	±2.0
V_IAT_ (km/h)	14.0	±0.6	15.1	±1.0

V_IAT_ - velocity at individual anaerobic threshold,

VO_2max_ - maximal oxygen consumption.

**Table 2:** Concentrations of conventional clinical chemical plasma parameters at different time points of the first exercise study.

	pre-run	0 h (immediately after) run	3 h after run	24 h after run
NEFA (µmol/L)	395±115	1276±392 *	330±254	282±142
Cortisol (nmol/L)	624±154	692±232	344±106 *	602±104
Somatotropin (µg/L)	1.13±1.8	13.7±8.2 *	0.1±0.09	0.05±0.1
Creatine kinase (U/L)	225±148	268±156	266±158	234±147
Epinephrine (nmol/L)	0.31±0.20	0.41±0.29	0.33±0.18	0.33±0.19
Norepinephrine (nmol/L)	1.88±0.71	4.28±1.37 *	2.38±1.09	2.43±0.78
Lactate (mmol/L)	0.91±0.37	(1.63±0.47 *)a)2.08±0.86 *	Nd	nd

Values are shown as mean ± SE.

a) measured after 10 min of treadmill performance.

*p<0.05 vs. pre.

nd = not determined.

### Non-targeted metabolomics analysis using reversed-phase UPLC-qTOF-MS

**Figure 1:**
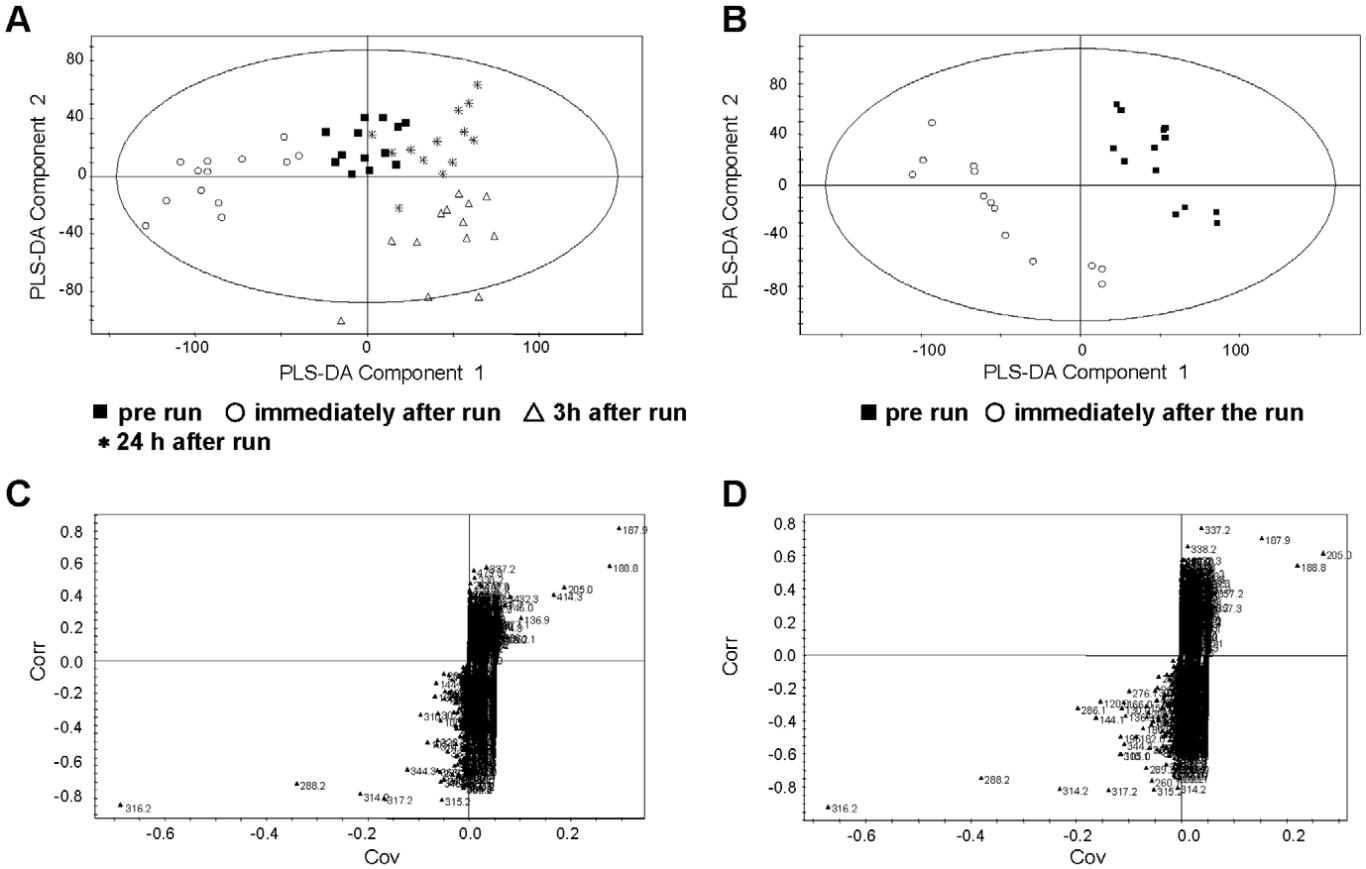
Identification of metabolites reflecting changes in the metabolite pattern during moderate intensity exercise and recovery. Comparison of the time-dependent changes in the plasma metabolome of 13 individuals after a 60 min treadmill run at 93% velocity at individual anaerobic threshold (V_IAT_; approximately 75% of VO_2max_), showing the metabolome at rest, immediately at the end of the physical activity and at two time points in the recovery phase. The analyses were performed by partial least squares-discriminant analysis (PLS-DA) and OSC-filtering. (A) OSC-filtered PLS-DA score plot showing pre-run (▪), immediately after run (○), three hours after run (Δ) and 24 hours after run (*); (B) OSC-filtered PLS-DA score plot showing pre run (▪) and immediately after run (○). (C,D) The corresponding S-plot to (A) and (B), respectively. The variables are labelled with *m/z* values. Potential metabolic biomarkers including the corresponding *m/z* values are presented in Table 3 and Table 4.

The plasma metabolite pattern of the first study group was analyzed by UPLC-qTOF-MS in the positive ionization mode at four time points, before the run, immediate afterwards (0 h), 3 h and 24 h after the run. We constructed a model using partial least squares-discriminant analysis (PLS-DA) with an orthogonal signal correction (OSC) data filter using randomized sample ordering, according to Wold et al. [24]. As indicated by the PLS-DA score plot, considerable changes of the metabolome in the plasma at each time point representing more than 2500 metabolite ions per individual are visible (Figure 1A). To ensure that the calculated model was reliable and the observed clustering was not due to chance, we performed an internal validation using 7-fold cross-validation as described in [25]. The estimated goodness of fit of R^2^Y was 0.926, and the goodness of prediction of Q^2^Y was 0.548 which underlines the robustness of the model. To assess the significance of the predictive ability, a response permutation test (Y scrambling) was used and showed no overfitting (R^2^Y-intercept of 0.490, and Q^2^ –intercept of -0.775). Moreover, a clear, distinct separation was obtained by generation of the OSC-PLS-DA score plot of the metabolome of the pre-exercise state and immediately after exercise (Figure 1B).

### Identification of metabolites reflecting changes in the plasma metabolite pattern during exercise and recovery

To mine the complex data and propose which metabolite ion masses could be of potential interest, we used the S-plot of SIMCA-P [26]. The S-plot given in Figure 1C shows the *m/z* values of the most altered metabolite ions in plasma which are responsible for the separation of the time points in the PLS-DA score plot in Figure 1A. The metabolite ions with the greatest influence on the cluster are located furthest away from the center of the S-plot. The characterization of the most discriminative plasma metabolite ions by mass spectrometric fragmentation pattern resulted in the identification of several medium chain acylcarnitine species namely, C8:0(octanoyl)-, C10:0(decanoyl)-, C10:1(decenoyl)-, C10:2-OH(hydroxy-decadienoyl)- and C12:0(dodecanoyl)-carnitine (Table 3). Since we used positive ionization for the mass spectrometric analysis, highly negatively charged metabolites such as fatty acids could not be detected in our approach.

**Table 3:** Most influential metabolites contributing to the separation of the clusters of the exercise and recovery phase shown in Figure 1A.

retention time	mass (m/z)	Metabolite	p-value(ANOVA)
15.83	432.3	Glycochenodeoxycholic acid*	0.066
19.59	337.2	Unidentified	0.006
6.62	105.0	Hippurate	0.014
12.48	288.2	C8:0 carnitine	0.001
14.30	314.2	C10:1 carnitine	<0.001
14.30	315.2	isotope of 314.2	<0.001
15.42	328.2	C10:2-OH carnitine	0.002
15.60	316.2	C10:0 carnitine	<0.001
15.60	317.2	isotope of 316.2	<0.001
18.76	344.2	C12:0 carnitine	<0.001

Only metabolites showing significant changes are given.

*mass (m/z) represents a fragment of this metabolite.

Identification of the discriminating ion masses responsible for the clear separation of the resting state and the acute exercise state (Fig. 1B,D) revealed again that acylcarnitines were the major metabolite group of exercise-induced biomarkers with increases in C6:0(hexanoyl)-, C8:0-, C10:0-, C10:1-, C12:0-, and C14:2(tetradecadienoyl)-carnitine (Table 4).

**Table 4:** Most influential metabolites contributing to the separation of the clusters of the exercise and pre run phase shown in Figure 1C.

retention time	mass (m/z)	Metabolite	immediately after run vs. pre run	p-value(t-test)
6.62	105.0	Hippurate	↑	0.054
8.94	260.1	C6:0 carnitine	↑	0.010
12.48	288.2	C8:0 carnitine	↑	0.019
12.48	289.2	isotope of 288.2	↑	0.019
14.30	314.2	C10:1 carnitine	↑	0.001
14.30	315.2	isotope of 314.2	↑	0.001
15.60	316.2	C10:0 carnitine	↑	<0.001
15.60	317.2	isotope of 316.2	↑	<0.001
18.38	368.2	C14:2 carnitine	↑	0.01
18.76	344.3	C12:0 carnitine	↑	0.012
19.59	337.2	Unidentified	↓	0.008

Only metabolites showing significant changes are given.

### Time-course of the acylcarnitine species plasma levels in the exercise phase and under recovery conditions

**Figure 2:**
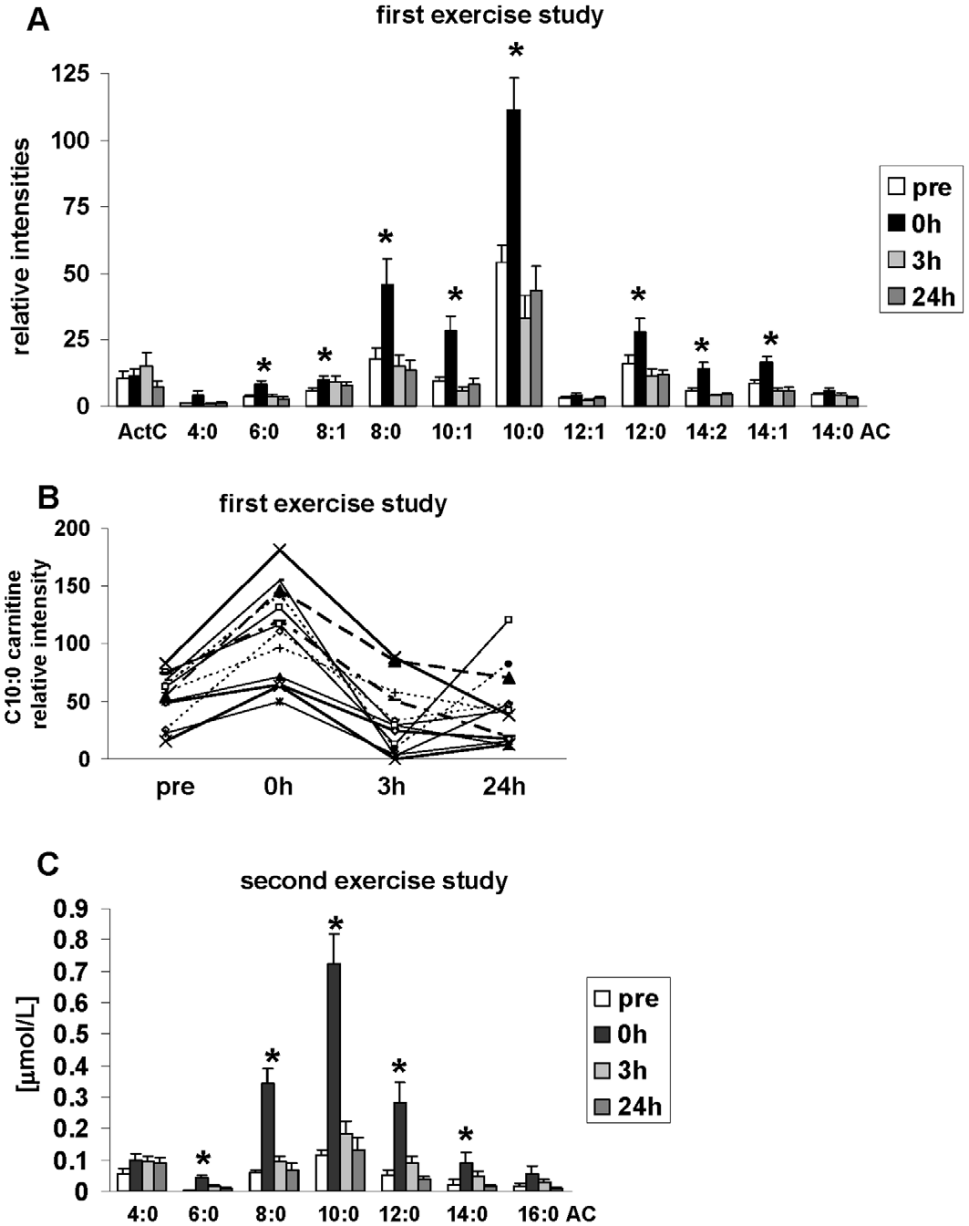
Time-course for the acylcarnitine species plasma levels during the exercise phase and under recovery conditions. (A) Time-dependent changes of acylcarnitine species during the exercise bout and in the recovery phase, based on non-targeted (NT-)metabolomics signal intensity data. The relative amounts of acylcarnitines are based on peak heights. Values are means ±SE. *p<0.05, significantly different from the pre run signal intensity; (*n = *13). (B) Time-dependent changes of 13 individual C10:0 carnitine levels based on NT-metabolomics data. (C) Quantitative analysis of time-dependent changes of plasma acylcarnitine concentrations investigated in eight individuals performing a continuous 120 min treadmill run at 70% V_IAT_ in an independent second exercise study. Values are means ±SE. *p<0.05, significantly different from the pre-run concentrations. As a C8- instead of a C18-reversed phase UPLC-column was used to achieve the detection of the long-chain C16 (palmitoyl)-carnitine, the analysis of C2:0-carnitine was not possible in this experiment.

The signal intensities (based on the peak height) of all acylcarnitines detectable in our mass spectrometric data showed similar kinetics, namely a strong, significant increase immediately after the exercise bout and a pronounced decrease thereafter leading to signal intensities similar to the pre-run data, except for C2:0(acetyl)-, C4:0(iso/butyryl)-, C12:1- and C14:0-carnitine, which showed no significant changes at any time point (Figure 2A). Exemplarily, the time course of C10:0-carnitine is presented in Figure 2B for each individual runner.

As we applied a non-targeted approach, the measured signals are only relative intensities. We verified the acylcarnitine kinetics quantitatively in a second exercise study. Here, the treadmill run lasted over 120 min at a lower intensity of 70% V_IAT_. Accordingly, blood lactate concentrations were not significantly increased after the run (1.49±0.66 vs. 1.23±0.37 mmol/L before run), while the increase in NEFA levels was comparable (872±407 vs. 112±78 µmol/L). Blood samples in the first study were drawn in the fasted state (followed by a standardized breakfast) and in the second investigation resting blood samples were collected following the assessment of IMCL by magnetic resonance spectroscopy, i.e. 1 h 45 min after a standardized breakfast. Despite these differences in study design, the kinetics of the absolute plasma concentrations of the acylcarnitines in the second study were completely comparable with the time courses found in the first study group (Fig. 2C). The sum of C8:0, C10:0, and C12:0-carnitine in the second study increased from 0.22 µmol/L before to 1.34 µmol/L immediately after the run. Determination of intramyocellular lipids revealed a significant decrease in intracellular lipid stores of soleus (reduction of 22±8%) and tibialis anterior muscles (reduction of 24±16%; data not shown), suggesting that intramyocellular lipids were at least partially metabolized during the 120 min treadmill run.

### ^13^C-acylcarnitine synthesis and release from human myotubes

**Figure 3:**
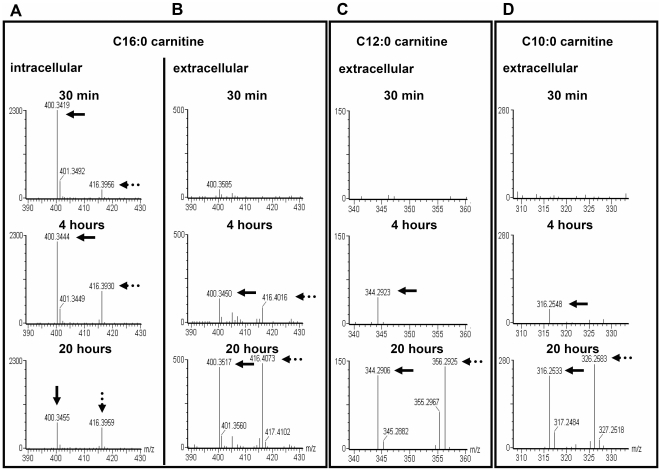
^13^C-Acylcarnitine synthesis and release from primary human myotubes. Comparison of the time course of intracellular (A) and extracellular (B) intensity levels of ^12^C- and ^13^C-palmitoyl-carnitine in an experiment with primary human myotubes incubated with a mixture of 125 µM [U-^13^C_16_]palmitate/125 µM palmitate for 30 min, 4 h and 20 h. (C) The time course of the ^12^C- and ^13^C-signal intensity of dodecanoyl-carnitine and (D) decanoyl-carnitine. The ^13^C-signal of the respective acylcarnitine is marked by a dash dotted arrow, the ^12^C-signal by a solid arrow.

To obtain direct proof that the working skeletal muscle could be the source of the elevated acylcarnitine concentrations in the plasma, we studied the time course of acylcarnitine metabolites derived from ^13^C-palmitate upon incubation with primary human myotubes. Intracellular and released acylcarnitines were determined by UPLC-qTOF-MS. After 30 min, 4 and 20 h of incubation with ^13^C-palmitate intracellular ^13^C-palmitoyl-carnitine was clearly present (Fig. 3A). After 4 and 20 hours of ^13^C-palmitate incubation ^13^C-palmitoyl-carnitine was also detectable extracellularly (Fig. 3B), and ^13^C-derivates of C12:0 and C10:0 carnitine were found after 20 h in the supernatant (Fig. 3 C,D). These data provide clear evidence that primary human myotubes rapidly metabolize palmitate to palmitoyl-carnitine, which is released in the supernatant together with C12:0- and C10:0-carnitine resulting from partial oxidation of palmitate. We did not detect other acylcarnitine species.

### Effects of acylcarnitines on fatty acid oxidation in primary human skeletal muscle cells and soleus, extensor digitorum longus, tibialis and quadriceps muscles of mice

**Figure 4:**
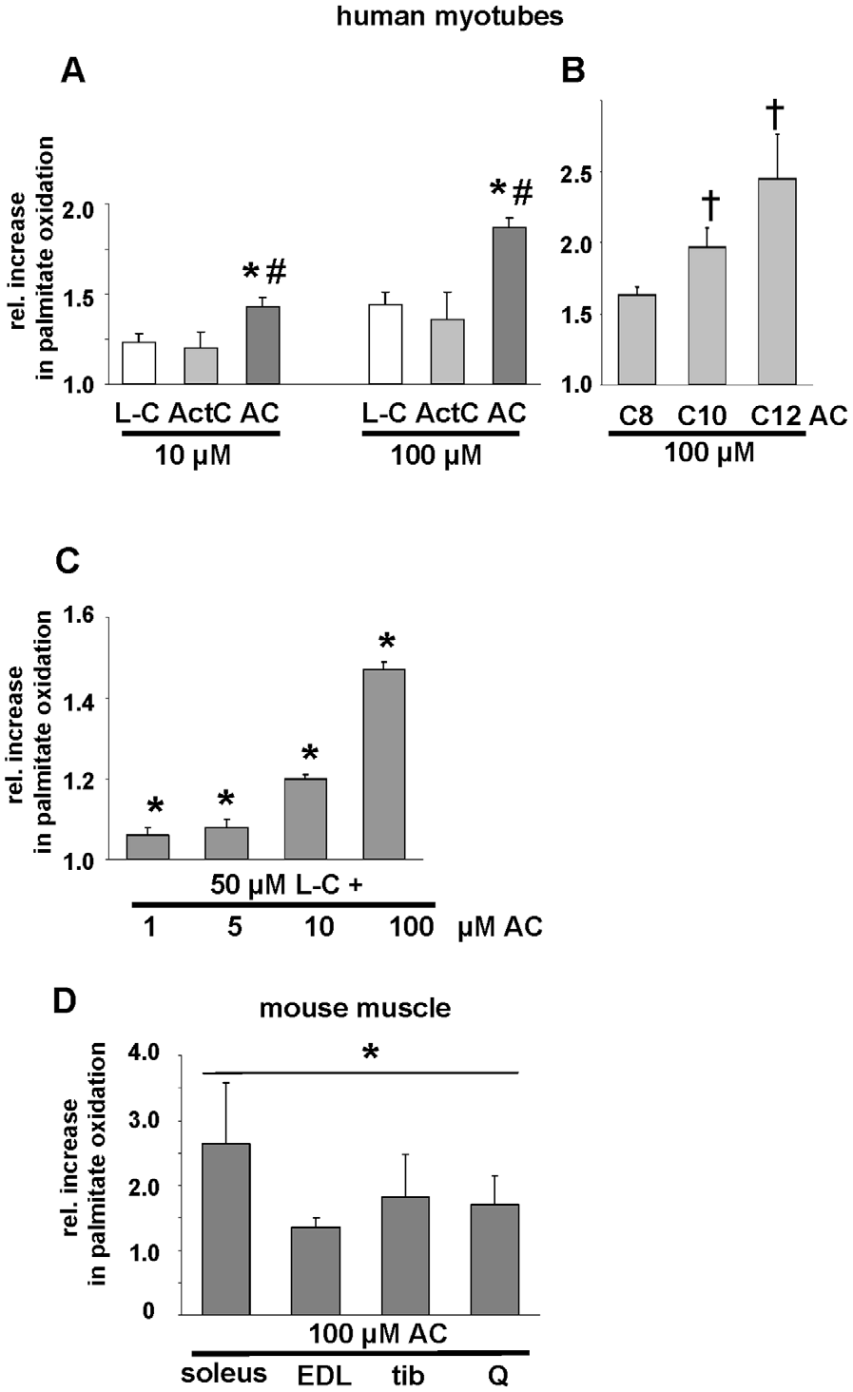
Effects of acylcarnitines on fatty acid oxidation in primary human skeletal muscle cells as well as soleus, extensor digitorum longus, tibialis and quadriceps muscles from mice. Oxidation of ^3^H-palmitate determined after 4 h in human myotubes in the presence of L-carnitine (L-C), acetylcarnitine (ActC), an equimolar mixture of C8:0-, C10:0- and C12:0-acylcarnitine (AC), or C8:0-(C8-AC), C10:0- (C10-AC) and C12:0-acylcarnitine (C12-AC) as indicated (A,B). Values of ^3^H-palmitate oxidation of control cells were set as 1. (C) Palmitate oxidation in the presence of 50 µM L-carnitine and 1, 5, 10 or 100 µM AC. Values of ^3^H-palmitate oxidation of cells solely incubated with 50 µM L-carnitine were set as 1; shown are means ± SEM from 4 independent experiments, * p<0.05 vs. L-carnitine; # p<0.05 vs acetyl-carnitine; † p<0.05 vs. C8-AC or C10-AC, respectively. (D) Oxidation of ^3^H-palmitate in mouse soleus, extensor digitorum longus (EDL), tibialis (tib) or quadriceps (Q) muscle from three different mice determined after 90 min in the presence of an equimolar mixture of C8:0-, C10:0- and C12:0-acylcarnitine as indicated. Shown are means ± SEM, values of ^3^H-palmitate oxidation in untreated tissues were set as 1, * p<0.05 vs. untreated tissues.

To study the potential effect of medium chain acylcarnitines on fatty acid metabolism, human myotubes were incubated with an equimolar mixture of C8:0-, C10:0- and C12:0-carnitines (in the following referred to as (C8-12:0) carnitines). Comparison of palmitate oxidation in the presence of 10 or 100 µM L-carnitine, acetyl-carnitine or (C8-12:0) carnitines revealed that the medium chain acylcarnitines were most efficient in supporting β-oxidation (Fig. 4A), with C12:0 carnitine being more potent than C10:0- and C8:0-carnitine (Fig. 4B). When we performed palmitate oxidation in the presence of 50 µM L-carnitine and added increasing concentrations of (C8-12:0) carnitine we found a slight, but significant increase in fatty acid oxidation even in the presence of 1 µM of medium chain acylcarnitines (Fig. 4C), which is close to the detected physiological plasma concentration of 1.34 µM of (C8-12:0) carnitine after the run. The additional effect of 10 or 100 µM (C8-12:0)-acylcarnitine (Fig. 4C), which may reflect local extracellular concentrations in skeletal muscle under exercise conditions, was comparable to the difference in palmitate oxidation between equimolar concentrations of L-carnitine and (C8-12:0)-acylcarnitine in figure 4A. To further verify the activation of palmitate oxidation in entire muscle, the effect of 100 µM (C8-12:0)-acylcarnitines was studied ex vivo in oxidative soleus muscle as well as in glycolytic extensor digitorum longus, tibialis and quadriceps muscles from mice. A comparable increase of palmitate oxidation as detected in human myotubes was found (Fig. 4D) supporting a physiological function of the (C8-12:0)-carnitines in skeletal muscle.

### Gene expression in primary human skeletal muscle cells after treatment with acylcarnitines

**Figure 5:**
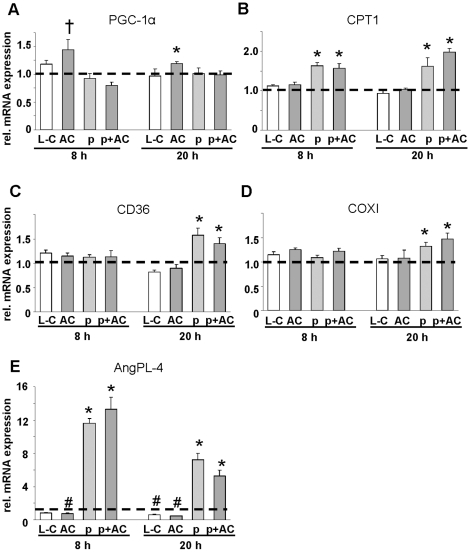
Gene expression in primary human skeletal muscle cells after treatment with acylcarnitines. mRNA expression of PGC-1α (A), CPT1b (B), CD36 (C), COX1 (D) and AngPL-4 (E) related to β-actin mRNA levels after incubation with 100 µM L-carnitine (L-C), an equimolar mixture of C8:0-, C10:0- and C12:0-acylcarnitine (total 100 µM; AC), 60 µM palmitate (p), or p and AC as indicated. Values are shown as fold changes compared with untreated (L-C, AC) or BSA-treated (p, p+AC) control cells, shown are means ± SEM; * significant increase with p<0.05 vs. control cells, † p = 0.06 vs. control cells; # significant decrease with p<0.05 vs. control cells. The broken black line indicates the 1-fold expression in control cells.

One effect of acylcarnitines regulating fatty acid metabolism in human myotubes could be altered expression of key regulators of fatty acid transport or oxidation or lipolysis. We compared the effect of 100 µM (C8-12:0)-acylcarnitine with 100 µM L-carnitine, 60 µM palmitate and the combination of acylcarnitine and palmitate on the expression of peroxisome proliferator activated receptor-γ coactivator (PGC)-1α, carnitine palmitoyltransferase (CPT)1b, CD36, cytochrome c oxidase subunit I (COX1), and angiopoietin-like (AngPL)-4 after 1, 3, 8 and 20 h of treatment. After short term incubation for 1 or 3 h we found no effects of acylcarnitines (data not shown), while after 8 and 20 h a slight increase in PGC-1α expression was detected (Fig. 5A). The induction of CPT1, CD36, COX1 and AngPL-4 expression by palmitate was not influenced by acylcarnitines (Fig. 5B–E), while acylcarnitines alone decreased AngPL-4 expression (Fig. 5E). Thus, the mRNA expression data could not explain the effect of acylcarnitines on palmitate oxidation.

## Discussion

In our exercise studies under moderate intensity, predominantly aerobic conditions we found an up to nine-fold increase in medium chain and long chain acylcarnitines immediately after running, which decreased very rapidly in the recovery phase. The major findings are: firstly, medium chain C8:0-, C10:0-, and C12:0-carnitine are dominating exercise biomarkers in the plasma metabolite pattern immediately after exercise vs the pre-exercise state; secondly, primary human skeletal muscle cells can produce and release acylcarnitines using palmitate as a substrate; and thirdly, C8:0-, C10:0-, and C12:0-carnitine support palmitate oxidation in skeletal muscle cells and muscle tissue.

The identification of exercise-related biomarkers with metabolic properties is key to understanding the beneficial effects of physical activity. In our non-hypothesis-driven metabolic profiling approach within more than 2500 metabolite ions per individual, we found that medium and long chain acylcarnitines are the most discriminating exercise-related metabolites under the applied physiological and analytical conditions. The specific and marked elevation of medium and long chain acylcarnitine pattern was dominated by C8:0-, C10:0-, and C12:0-carnitine. Since a pronounced decrease of plasma acylcarnitine levels during an oral glucose tolerance test was recently reported suggesting that food intake results in decreased acylcarnitine levels [16], breakfast-related effects can be exclude in our studies. However, in the recovery phase it is quite likely that not only the end of the exercise activity but also the food consumption thereafter could be involved in the decline of acylcarnitines.

The exercise-related increase in plasma acylcarnitine concentrations in humans had first been described in the 1980s and early 1990s [27]–[30]. Interestingly, after high-intensity or exhaustive exercise with marked plasma lactate concentrations above 5 mM plasma acylcarnitine concentrations failed to increase, while elevated acetylcarnitine levels were found [27], [28]. We found in both studies with moderate exercise intensities and only slight or no detectable increases in plasma lactate pronounced increase in acylcarnitines, while acetylcarnitine did not change significantly. Since the major source of acetylcarnitine is acetyl-CoA formed from pyruvate oxidation [31], increases in acylcarnitines during exercise may indicate high rates of fatty acid oxidation and low reliance on glycolysis, which is supported by the moderate or lacking increase in lactate in our studies. Since based on methodological limitations in the 1980s and 1990s acylcarnitines had only been described as the difference between total and free carnitine [29], [30] or as short chain (≤10 carbons)- and long-chain (>10 carbons)-carnitines [27], [28]. The specification of medium chain acylcarnitines as exercise-increased biomarkers was not possible in these earlier studies.

The working muscle has always been considered as the source of acylcarnitines found in plasma during exercise conditions [28]–[30], and intramuscular acylcarnitine accumulation has been demonstrated in conditions of increased muscle fat oxidation [32]. In the present study we provide direct evidence for the ability of primary human myotubes to produce and release acylcarnitines from palmitate, indicating incomplete oxidation of this fatty acid. Our findings were also supported by a very recent study showing that a 24 h load of human myotubes with 1000 µM fatty acids and carnitine increases production and efflux of acylcarnitines [33].

The formation of acylcarnitines may serve as a “buffer” for the limited and metabolically important CoA pool [27], and has been viewed as a detoxifying system that permits mitochondrial efflux of excess acyl groups [34] leading to increased levels of acylcarnitines in blood and urine, well known from inborn mitochondrial diseases [35], [36]. Plasma acylcarnitines were also increased in high fat diet-induced obesity in animal models [32], [33] and in obese and type 2 diabetic humans [37], suggesting that efflux of acylcarnitines occurs when the influx of acyl-CoA into mitochondria exceeds the capacity for complete oxidation of the fatty acids due to chronic oversupply with substrate. However it is important to note that increased availability of substrates for β-oxidation is also a physiological phenomenon associated with the high lipolytic rates found during exercise or starvation, subsequently resulting in acute efflux of acylcarnitines into plasma depicted as total esterified carnitines [29], [30] or total long chain acylcarnitines [38].

What we believe is one major finding of the present study is the induction of higher rates of palmitate oxidation in primary human myotubes and soleus, extensor digitorum longus, tibialis and quadriceps muscle from mice in the presence of extracellular C8:0-, C10:0-, and C12:0-carnitine. In muscles from mice stimulation of palmitate oxidation is greatest in murine soleus muscle which is predominantly type I fibers (oxidative) and least in EDL which is primarily type IIb (glycolytic) fibers. This finding would be the predicted result if the effect of medium-chain acylcarnitines is primarily via its impact on oxidative metabolism. Free carnitine availability has been considered to be the limiting factor for muscle fat oxidation for decades and manipulating the L-carnitine pool in the muscle by dietary regiments is still the focus of scientific and commercial approaches to enhance β-oxidation [33]. Of note, the direct comparison of the effect of C8:0-, C10:0-, and C12:0-carnitine and L-carnitine on palmitate oxidation revealed higher rates of palmitate oxidation in the presence of acylcarnitines. In this experimental setting, human myotubes were not incubated with additional L-carnitine which raises the question of the physiological relevance of this finding. Plasma levels of free carnitine are 30–50 µM with no significant changes during moderate intensity exercise [27], [28], [30]. Thus one could argue that the myotubes are in a state of L-carnitine deficiency, and providing L-carnitine during palmitate oxidation clearly increased fatty acid oxidation. But, the effect of equimolar concentrations of medium chain acylcarnitines on palmitate oxidation was even stronger. Moreover, when palmitate oxidation was performed with physiological concentrations of L-carnitine (50 µM) and acylcarnitines (1 µM) we found a slight, but significant effect on fatty acid oxidation. Therefore we assume that one biological function of medium chain acylcarnitines could be to support lipid oxidation during exercise. In addition, since during exercise skeletal muscle free carnitine content is reduced to approximately 25%, it could be speculated that the working muscle in vivo might also be in a state of L-carnitine deficiency [27], [31], [39].

How can medium chain acylcarnitines support oxidation of palmitate? It is possible that the transport rate of acylcarnitines into the cell is higher than the rate for L-carnitine, which is saturated in the basal state. The K_m_ for carnitine of the organic cation transporter OCTN2 is in the low µmolar range (4.3 µM in vitro; [40]). Acylcarnitines have the potency to inhibit the transport of L-carnitine by OCTN2 with a more pronounced effect with increasing chain length [41], [42]. Since the increasing effect of acylcarnitines on palmitate oxidation also depends on the length of the acyl-moiety with C12 being more effective than C10 and C10 more than C8, it could be speculated that the acylgroup facilitates the transport of carnitine across the cell membrane although it needs to be confirmed whether OCTN2 is the responsible transporter. Moreover, acylcarnitines can enter the mitochondria directly via carnitine acylcarnitine translocase [43] without the need for activation by acyl-CoA synthetase (ACS) or the the carnitine transporter system with CPT1 as the major site of control of lipid oxidation. Once inside the mitochondrial matrix, acylcarnitines are transesterified back to free carnitine and the corresponding acyl-CoA in a reaction catalysed by CPT2. This may lead to an increased ATP production since they bypass the β-oxidation enzymes specific for LCFAs and are directly metabolized via medium chain acyl-CoA dehydrogenase followed by the TCA-cycle. This increase in ATP may favour the ACS-catalyzed reaction relevant for the activation of palmitate [44]. As outlined above, the release of free carnitine in the CPT2-catalyzed reaction may increase the carnitine pool supporting the CPT1-catalyzed formation of palmitoyl-carnitine, thereby increasing the flux of palmitate into the mitochondria.

The concept that medium chain acylcarnitines per se act as signalling molecules thereby activating pathways involved in increased β-oxidation can also not be excluded. Acetyl-carnitine at high concentration (1 mM) has been shown to activate AMP-activated kinase [45]. We found slight increases in PGC-1α expression by medium chain acylcarnitines, which might indicate activation of AMPK. However, we could not detect increased phosphorylation of AMPK or its substrate acetyl-CoA carboxylase after treatment with medium chain acylcarnitines in our cell culture experiments with primary human myotubes (data not shown). Thus the signalling properties of acylcarnitines remains speculative, but the fact that acylcarnitines can bind to phospholipid bilayers, as do other amphipathic lipids, and thereby alter the function of membranes or membrane-bound proteins is also worth taking into consideration [46].

In conclusion, increased plasma levels of medium chain acylcarnitines are not only dominating biomarkers of moderate intensity exercise, one of the most important and successful interventions for improving health benefits, but might be also biologically active molecules enhancing β-oxidation.

## Materials and Methods

### Ethics Statement, subjects and study design

The protocol (74/2004) was approved by the Institutional Review Board of the University of Tuebingen, Schleichstr. 8, 72076 Tuebingen (board chairman: Prof. Dr. D. Luft) according to the Declaration of Helsinki, and all subjects gave written informed consent before the study commenced. The investigation was conducted in accordance with the ethical principles of Good Clinical Practice. A total of twenty-one healthy male subjects gave informed consent to participate in the study. All subjects had to participate in a preliminary testing procedure one week prior to the main exercise tests. Running velocity at the individual anaerobic threshold (V_IAT_) was determined with an incremental exercise test (start 6 km·h^−1^, increment 2 km·h^−1^ every 3 min) on the treadmill (Saturn, HP Cosmos, Traunstein, Germany) (Table 1). V_IAT_ was assessed as described by [47]. Maximal oxygen consumption (VO_2max_) was assessed during an additional ramp test (start 8 km·h^−1^ increment 1 km·h^−1^ every 0.5 min) until exhaustion [48].

In the first exercise experiment, thirteen of them were subjected to a 60 min continuous run of moderate intensity. Treadmill speed was adjusted to 93% of the V_IAT_ as assessed in the preliminary testing procedure. This corresponds to approximately 75% of VO_2max_. Resting blood samples (pre-run) were drawn in sitting position after an overnight fasting period at 8:15 am. Immediately after blood sampling, the subjects received a standardized breakfast (two small bread rolls; total energy content 225 kcal) and exercise was started at 9:15 am. Further blood samples were drawn in sitting position immediately (0 h), 3 and 24 h after the end of the exercise bout. In a second investigation eight subjects completed a 120 min continuous treadmill run at a running velocity of 70% of the V_IAT_(corresponding to approximately 55% VO_2max_). Here, resting blood samples were drawn in sitting position direct before the run which began after the measurement of intramyocellular lipids by MRS (see below), i.e. 1 h 45 min after a standardized breakfast (two small bread rolls; total energy content 225 kcal). In the recovery phase food consumption was not restricted in both studies.

### Plasma parameters

Glucose, total nonesterified fatty acids (NEFA) and creatine kinase activity (CK) were measured by the ADVIA 1650 clinical chemical analyzer; cortisol and somatotropin were analyzed with the ADVIA Centaur immunoassay system (both Siemens Healthcare Diagnostics, Fernwald, Germany). The concentrations of epinephrine and norepinephrine were determined by HPLC using an analytical kit (Chromsystems, Munich, Germany). Capillary blood for lactate measurements was obtained from the earlobe and measured with the EBIO system (Eppendorf, Hamburg, Germany).

### Determination of intra-myocellular lipids (IMCL) by magnetic resonance spectroscopy

Localized image guided proton magnetic resonance spectra of the tibialis anterior muscle and the soleus muscle were acquired on a 1.5-Tesla whole body imager (Magnetom Vision, Siemens, Erlangen, Germany). For volume selection, a single voxel STEAM technique was applied. Measurement parameters were: echo time  = 10 msec, repetition time  = 2 sec, volume of interest 11×11×20 mm^3^, 40 acquisitions. IMCL were quantified as previously described [49].

### Cell culture experiments

Human primary skeletal muscle cells were cultured and differentiated as previously described [50]. To study the metabolism of palmitate to carnitine derivates, myotubes were incubated with 125 µM [U-^13^C_16_]palmitate (99 atom % ^13^C) and 125 µM palmitate for 30 min, 4 h and 20 h in α-MEM containing 5.5 mM glucose and 2% fetal bovine serum.

For palmitate oxidation, myotubes were incubated in α-MEM containing 5.5 mM glucose, 0.1% fatty-acid free BSA, 2 µCi/ml [9,10-^3^H(N)]-palmitate (PerkinElmer, Rodgau-Jügesheim, Germany) and 60 µM unlabelled palmitate for 4 h. L-carnitine, acetylcarnitine or an equimolar mixture of C8:0-, C10:0- and C12:0-carnitines (in the following referred to as (C8-12:0) acylcarnitines) was added as indicated. For palmitate oxidation in muscle tissue, muscles were removed immediately after decapitation of mice and incubated for 90 min at 37°C in the presence or absence of (C8-12:0)-carnitines as described above. Production of tritiated water was measured after solid-phase extraction of the supernatant using Oasis HLB cartridges (Waters, Milford, MA) in a scintillation counter.

### RT-PCR and Real-time quantitative PCR analysis

For RNA expression, myotubes were incubated in α-MEM containing 5.5 mM glucose and palmitate, L-carnitine and acylcarnitines were added as indicated. Reverse transcription of total RNA (1 µg) was performed in a volume of 20 µl using random hexamers and Avian Myeloblastosis Virus reverse transcriptase with the First strand cDNA synthesis kit for RT-PCR (Roche, Mannheim, Germany). Aliquots (2 µl) of the reverse transcription reactions were then submitted in duplicate to online quantitative PCR with the Light Cycler system (Roche, Mannheim, Germany) with SYBR® green using the FastStart DNA-MasterSYBR Green I (Roche, Mannheim, Germany). The following primer pairs were used: human PGC-1α sense: tgtggaactctctggaactg, antisense: tgaggacttgctgagtggt, product of 232 bp; human CPT1b sense: ctcctttccttgctgaggtg; antisense: tctcgcctgcaatcatgtag, product of 177 bp; human CD36 sense: ctaatgccagttggagacct, antisense: actgtgaagttgtcagcctc, product of 335 bp; human AngPL-4 sense: agcatctgcaaagccagttt, antisense: gcgcctctgaattactgtcc, product of 278 bp; human COX1 sense: ggcctgactggcattgtatt, antisense: tggcgtaggtttggtctagg, product of 177 bp; human β-actin sense: gagcaagagaggcatcctca, antisense: agcctggatagcaacgtaca, product of 238 bp. The PCR was performed in a volume of 20 µl: 2 µl FastStart DNA-MasterSYBR Green I, MgCl_2_ 4 mmol/l, and primers according to a primer concentration of 1 µmol/l. The instrument settings were: After denaturing at 95°C for 10 minutes, cycling was performed by denaturing at 95°C for 15 s, annealing at 65°C for 10 s, elongation for 10 s for PGC-1α, the number of cycles was 45; annealing at 68°C for 10 s, elongation for 13 s for CPT1b, the number of cycles was 45; annealing at 64°C for 10 s, elongation for 14 s for CD36, the number of cycles was 45; annealing at 68°C for 10 s, elongation for 11 s for AngPL-4; the number of cycles was 45; annealing at 55°C for 10 s, elongation for 10 s for COX1, the number of cycles was 50; annealing at 67°C for 10 s, elongation for 11 s for β-actin, the number of cycles was 40.

### UPLC-qTOF-MS analysis

#### Sample preparation for UPLC-qTOF-MS analysis

Plasma samples were prepared as described elsewhere [51] and cell culture supernatants accordingly. For cell lysates, cells were trypsinized, lyzed, deproteinized with acetonitrile, run to dryness in a vacuum centrifuge, stored at −20°C, and reconstituted in 150 µl acetonitrile and water (8∶2). The quantitative analysis of acylcarnitine-species in plasma was performed by the addition of isotope labeled internal standards ([8,8,8 ^2^H_3_] octanoyl-L-carnitine, [10, 10, 10 ^2^H_3_] decanoyl-L-carnitine and [12, 12, 12 ^2^H_3_] dodecanoyl-L-carnitine (Organic Synthesis Unit, VU Medical Center, Amsterdam; The Netherlands) to the samples. Standard curves were established using plasma added isotope label standard at concentrations from 0.01 to 1 nmol/ml.

### UPLC analysis

The chromatographic separation of plasma from the first exercise study was performed on a 100×2.1 mm ACQUITY 1.7 µm/C18 column using an ACQUITY-UPLC system (Waters Corp, Milford, USA). The column was maintained at 30°C and the gradient program at a flow rate of 0.25 ml/min was 100% A (0.1% formic acid in water) for 1 min, changed to 100% B (acetonitrile) linearly within 30 min and held for 4 min, finally back to 100% A. Plasma samples from the second study as well as skeletal muscle cell lysates and supernatants were investigated using a 100×2.1 mm ACQUITY 1.7 µm/C8 column maintained at 35°C applying a gradient program at a flow rate of 0.35 ml/min starting with 90% A (0.1% formic acid in water) for 0.5 min, changed to 100% B (acetonitrile) linearly within 24 min and held for 4 min, finally back to 90% A.

### Mass spectrometric procedures and data collection

The UPLC system was coupled to a Micromass qTOF-MS (Manchester, UK) equipped with an electrospray source operating in positive ion mode (full scan mode from *m/z* 100-650) adjusted to the following settings: source temperature 120°C, cone gas flow of 50 l/h, desolvation gas temperature 300°C, desolvation gas flow of 500 l/h, capillary voltage 3100 V, cone voltage to 35 V, scan time 0.4 s (using inter-scan delay of 0.1 s), and collision energy 4 eV (collision gas: argon). All analyses were acquired using the lock spray to ensure accuracy and reproducibility. Leucine enkephalin was used as the lock mass (lock spray frequency: 20 s). Potential biomarkers were identified following our recently published analytical strategy for the identification of biomarkers in metabolomics studies, described in detail in [51].

Mass spectra were digitally analyzed using the Micromass MarkerLynx Applications Manager version 4.0 (Waters Ltd). The statistical calculation was performed using the intensity of the metabolite ions. The data were combined into a single matrix by aligning peaks with the same mass-retention time pair together from each data file in the data set. The intensity for each peak was normalized to the sum of the peak intensities for each data set to enable the comparison of the relative mass intensities of metabolites between the different data files.

### Data analysis

The pre-processed UPLC-qTOF-MS data were exported into Soft Independent Modeling of Class Analogy (SIMCA)-P (version 11.0, Umetrics AB, Umea, Sweden) for analysis and visualization by multivariate statistical methods. The “80% rule” [52], [53] was used to remove missing values, the variables having more than 80% non-zero measurement value in one or more of the groups were kept in the peak list. In total 2543 variables were merged to the matrix for the following data analysis. Furthermore, after Pareto scaling and OSC-filtering according to Wold et al. [24], partial least squares-discriminant analysis (PLS-DA) was applied. The S-plot was used to identify metabolites exerting a major influence on the group membership [26]. The predictive ability of the model was assessed by internal validation using 7-fold cross-validation and response permutation testing. Clinical chemical, anthropometric data of the individuals and cell culture experiments were computed using the statistical software packet JMP (SAS Institute, Inc., Cary, NC). p<0.05 was considered significant.
